# Gene Expression and Methylation Analysis in Melanomas and Melanocytes From the Same Patient: Loss of NPM2 Expression Is a Potential Immunohistochemical Marker for Melanoma

**DOI:** 10.3389/fonc.2018.00675

**Published:** 2019-01-21

**Authors:** Susumu Fujiwara, Hiroshi Nagai, Haruki Jimbo, Naoe Jimbo, Tomoyo Tanaka, Masukazu Inoie, Chikako Nishigori

**Affiliations:** ^1^Division of Dermatology, Department of Internal Related, Kobe University Graduate School of Medicine, Chuo-ku, Japan; ^2^Department of Diagnostic Pathology, Kobe University Graduate School of Medicine, Chuo-ku, Japan; ^3^R&D Department, Japan Tissue Engineering Co., Ltd, Gamagori, Japan

**Keywords:** melanoma, melanocyte, nevi, epigenetics, methylation, NPM2

## Abstract

DNA methylation is considered the primary epigenetic mechanism underlying the development of malignant melanoma. Since DNA methylation can be influenced by environmental factors, it is preferable to compare cancer and normal cells from the same patient. In order to compare the methylation status in melanoma tissues and melanocytes from the same individuals, we employed a novel epidermal sheet cultivation technique to isolate normal melanocytes from unaffected sites of melanoma patients. We also analyzed primary and metastatic melanoma samples, three commercially available melanocytes, and four melanoma cell lines. Cluster analysis of DNA methylation data classified freshly isolated melanomas and melanocytes into the same group, whereas the four melanoma cell lines were clustered together in a distant clade. Moreover, our analysis discovered methylation at several novel loci (*KRTCAP3, AGAP2, ZNF490*), in addition to those identified in previous studies (*COL1A2, GPX3*); however, the latter two were not observed in fresh melanoma samples. Subsequent studies revealed that *NPM2* was hypermethylated and downregulated in melanomas, which was consistent with previous reports. In many normal melanocytes, NPM2 showed distinct immunohistochemical staining, while its expression was lost in malignant melanoma cells. In particular, intraepithelial lesions of malignant melanoma, an important challenge in clinical practice, could be distinguished from benign nevi. The present findings indicate the importance of using fresh melanoma samples, not melanoma cell lines and melanocytes in epigenetic studies. In addition, NPM2 immunoreactivity could be used to differentiate melanomas from normal melanocytes or benign disease.

## Introduction

Malignant melanoma is one of the most fatal skin cancers, and continues to increase in prevalence (1). Various epigenetic changes—such as DNA methylation and histone acetylation—are associated with melanocyte carcinogenesis (2). Since DNA methylation levels are influenced by various environmental factors, such as ultraviolet radiation exposure or lifestyle (3), it seems preferable to assess epigenetic changes in melanomas and adjacent normal melanocytes from the same patient. Since minor environmental shifts—such as replenishing culture medium—can cause changes in methylation state (4), it is critical to limit experimental steps between cell isolation and analysis for methylation analysis. In contrast, previous studies have compared established melanoma cell lines or melanoma tissues with melanocytes from neonatal foreskins or commercially available melanocytes (5–8), and a comparative evaluation of cells derived from the same individual has not been performed to date, likely due to the difficulties in procuring enough cells required for analysis (9). In the present study, we utillized an epidermal sheet culture technique, capable of propagating a sufficient number of melanocytes, even from less proliferative tissue from elderly patients, and isolating melanocytes using a laser sorting technique, which enables the preparation of melanocyte and melanoma sample sets derived from the same patients for differential analysis of gene expression and methylation. Notably, our analyses revealed several genes showing differences in the methylation status between melanoma cell lines and fresh melanoma samples that emphasize the importance of assessing epigenetic changes in normal and malignant tissues derived from the same patient. Among these genes, we found that the loss of NPM2 could be a candidate immunohistochemical marker for differentiation of melanoma and melanocytes.

## Materials and Methods

### Tissue Samples and Cultured Cell Lines

Melanoma and normal skin samples were obtained from surgically resected tumor tissues (*n* = 7) and adjacent tissue or abdominal skin (*n* = 5), respectively, from patients at Kobe University Hospital. All participants provided written informed consent. Study procedures conformed to the Guidelines of Ethics Committee on Life Sciences and Genetic Analysis and were approved by the Institutional Review Board at the Kobe University Graduate School of Medicine (No. 69).

The SK-mel-28, G361, and DEOC-1 (10) melanoma cell lines were purchased from RIKEN BioResource Center (Tokyo, Japan) and cultured in Eagle's minimal essential medium supplemented with 10% fetal calf serum. The HM3KO melanoma cell line was established in our institution (11). Normal human epidermal melanocyte (NHEM) lines 1-6 were purchased from Kurabo (Osaka, Japan) and maintained in DermaLife medium (Kurabo) for less than five passages. NHEM 4-6 was newly used because all NHEM 1-3 with a small number of passages was exhausted. All melanoma cell lines, melanoma tissues, and melanocyte cell strains, as well as the clinicopathological characteristics of each patient, are shown in Table 1.

**Table 1:** Melanocyte and melanoma samples.

**Source**		
○PM1	PatientA	60 s, left shoulder, SSM
ΔPM2	PatientB	80 s, left cheek, NM
□PM3	PatientC	60 s, left sole, ALM
PM5	PatientE	80 s, lower leg, NM
PM6	PatientF	80 s, left cheek, LMM
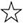 MM4	PatientD	60 s, vagina in transit metastasis
MM5	PatientE	From PM5 patient, inguinal node metastasis
○N1	PatientA	From PM1 patient, abdominal skin
○N1–2	PatientA	From PM1 patient, left shoulder
ΔN2	PatientB	From PM2 patient, left cheek
□N3	PatientC	From PM3, abdominal skin
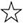 N4	PatientD	From MM4, abdominal skin
CM1		SK-mel 28
CM2		G361
CM3		DEOC-1
CM4		HM3KO
CN1		NHEM 1, neonatal donor
CN2		NHEM 2, adult donor
CN3		NHEM 3, adult donor
CN4		NHEM 4, neonatal donor
CN5		NHEM 5, neonatal donor
CN6		NHEM 6, adult donor

*PM, primary melanoma; MM, metastatic melanoma; CM, cultured melanoma cell line; N, normal melanocyte; CN, commercialy available normal melanocyte. ○Δ□
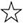
means from the same individual*.

### Isolation of Normal Melanocytes From the Tissues of Patients With Melanoma

Tissue samples were cultivated according to Green's method as previously reported (12, 13). Briefly, the normal skin specimens were cleaned to remove subcutaneous tissues, minced, and digested with trypsin to form a single cell suspension. The dissociated cells were then cultured with lethally X-ray-irradiated 3T3-J2 feeder cells to form pigmented epidermal sheets, primarily consisting of keratinocytes and melanocytes. These sheets were expanded to over 150 cm^2^, and trypsinized to form a single cell suspension for fluorescence-assisted cell sorting (FACS). Melanocytes were isolated from the mixed cell suspension with a MoFlo XDP (Beckman Coulter, Brea, CA) based on c-kit-APC antibody (CD117-APC, Beckman Coulter, Brea, CA) staining or an antibody-free method according to forward scatter and 670-nm emission elicited with 642 nm semiconductor laser. Purified cells were subsequently cultured in DermaLife medium.

### Extraction of DNA and RNA From Melanoma and Melanocytes

Genomic DNA and RNA were extracted from tumor tissues, isolated melanocytes, and cultured cells using an AllPrep DNA/RNA Micro Kit (QIAGEN, Hilden, Germany) according to the manufacturer's protocol.

### DNA Methylation and Expression Analysis

Methylation status was analyzed by microarray analysis (Infinium™ Human Methylation 450K Bead Chip, Illumina, San Diego, CA), which covers 99% of RefSeq genes with an average of 17 CpG sites per loci. Normalization for dye compensation was performed by subtracting the background value from the signal intensity of negative control using GenomeStudio software (Illumina). For normalization between samples, the quantile method in R software version 2.7.1 or version 3.5.1 (R Foundation for Statistical Computing, http://www.R-project.org) was used. Differences in methylation status are shown as the absolute difference between β values of multiple CpGs within the gene of interest (Table 2).

**Table 2:** Hypermethylated and Hypomethylated genes in melanoma compared to melanocyte.

**Gene name**	**Methylation β-value difference/1CpG site**
KRTCAP3	0.589795656
PAX3	0.540276268
HSPB6	0.49859898
COMT	0.42878184
AGAP2	0.413220909
ZNF490	0.387573592
DNAJA4	0.373014238
UCN	0.370786288
TTC22	0.35227618
BMP4	0.351273952
CTBP1	−0.303068271
FAT3	−0.303348838
TDRG1	−0.304617334
SDPR	−0.306425357
AJAP1	−0.310068984
GRIK2	−0.323373108
S100A4	−0.324588112
GIMAP5	−0.328492102
GPR31	−0.329135843
MIR548A2	−0.331845485
NPM2	0.082966405

Global mRNA expression was also assessed in 5 melanomas (PM2, PM3, CM1, CM2, CM4) and 5 melanocyte samples (N2, N3, CN1, CN2, CN3) from one set of microarray analysis (Illumina HumanHT-12 v4 BeadChip). Normalization was performed by subtracting the average value of signal intensities from 700 negative controls (as the background value) using GenomuStudio software. For normalization between samples, the quantile normalization method in GeneSpringGX version12.5 (Agilent Technologies, Santa Clara, CA) was used.

Melanoma and melanocyte samples were compared using the averaged methylation level of CpGs across each gene or methylation status of all individual CpGs.

Microarray data were deposited in GEO (expression data: GSE 122907, methylation data: GSE 122909).

### Real-Time Quantitative Reverse Transcriptase Polymerase Chain Reaction Analysis of mRNA Expression

Total RNA was extracted by using the RNeasy Mini Kit (QIAGEN, Hilden, Germany). cDNA synthesis was performed using the PrimeScript® RT Master Mix (Perfect Real Time) (TaKaRa Bio, Shiga, Japan). Real time quantitative reverse transcriptase polymerase chain reaction (qRT-PCR) was performed using an Applied Biosystems 7500 real-time PCR system (Applied Biosystems, Foster City, CA) and TB Green® Premix Ex Taq™ II (Tli RNaseH Plus) (TaKaRa Bio) with the following primer sets purchased from Takara Bio: *NPM2*, 5′-CCAGCAACCAGGAGGACAAG-3′ (sense) and 5′-GGAGAAAGCTGCACTCCTACCAT-3′ (antisense), with glyceraldehyde 3-phosphate dehydrogenase (GAPDH) as the internal control. mRNA expression were normalized according to the internal GAPDH control, and the relative expression values were plotted. RT-PCR was performed with duplicated sample.

### NPM2 and Melan-A Immunohistochemistry

Melanomas and benign melanocytic nevi from archived surgical resection samples at Kobe University Hospital—including 67 melanocyte samples, 32 melanomas, 10 Spitz nevi, 10 Unna nevi, 10 Miescher nevi, and 13 Clark nevi—were analyzed for NPM2 and Melan-A expression by immunohistochemistry using the BOND-MAX system (Leica Biosystems, Wetzlar, Germany) (Table 3). Briefly, 4-μm thick formalin-fixed, paraffin-embedded tissue sections were incubated in 10% H_2_O_2_ for 30 min at 65°C for demelanization, followed by incubation in citric acid buffer, pH 6.0 for 10 min at 99–100°C for antigen retrieval, and subsequent incubation with a mouse polyclonal antibody, raised against a full-length human NPM2 protein (1:300 dilution; Abnova, Taipei, Taiwan), or pre-diluted mouse monoclonal Melan-A antibody (Abcam, Cambridge, UK) for 45 min at 20°C. Slides were washed in Bond Wash Solution (Leica Biosystems), treated with Post Primary Alkaline Phosphatase (AP) for 15 min at room temperature, and with Polymer AP for 15 min. Colorimetric detection was performed with Mixed Refine Red for 5 min or DAB coloring for 10 min. Positive immunoreactivity was defined as strong 50% nuclear staining at 400 × magnification. All slides were independently evaluated by two investigators. Disputed samples were re-evaluated with a multi-headed microscope to reach a final agreement.

**Table 3:** NPM2 immunohistochemical staining.

**(A) Including dermal lesion**	**Positive rate**	*P*-value (χ^2^-test)^*^
Melanoma (*n* = 32)	15.6% (5/32)	Control
Melanocyte (*n* = 67)	74.6% (50/67)	*P* < 0.001
Benign nevus (*n* = 42)	23.8% (10/42)	*P* = 0.39
Spitz nevus (*n* = 10)	22% (2/9)
Unna nevus (*n* = 10)	10% (1/10)
Miescher nevus (*n* = 10)	10% (1/10)
Clark nevus (*n* = 13)	46% (6/13)
**(B) Only epidermal lesion**	**Positive rate**	***P***-value (χ^2^-test)^*^
Melanoma *in situ* (*n* = 14)	28.6% (4/14)	Control
Melanocyte (*n* = 67)	74.6% (50/67)	*P* = 0.001
Clark nevus (*n* = 10)	80.0% (8/10)	*P* = 0.013

* *P-value was analyzed compared with melanoma*.

### Statistical Analysis

Statistical analyses were performed using R, version 2.7.1. or 3.5.1. Clustering analysis used the group average method. Immunohistochemical staining was analyzed via chi-square analysis with Yate's continuity correction. *P* < 0.05 was considered statistically significant.

## Results

### Propagation of Patient-Derived Melanocytes With an Epidermal Sheet Culture Method

**Figure 1:**
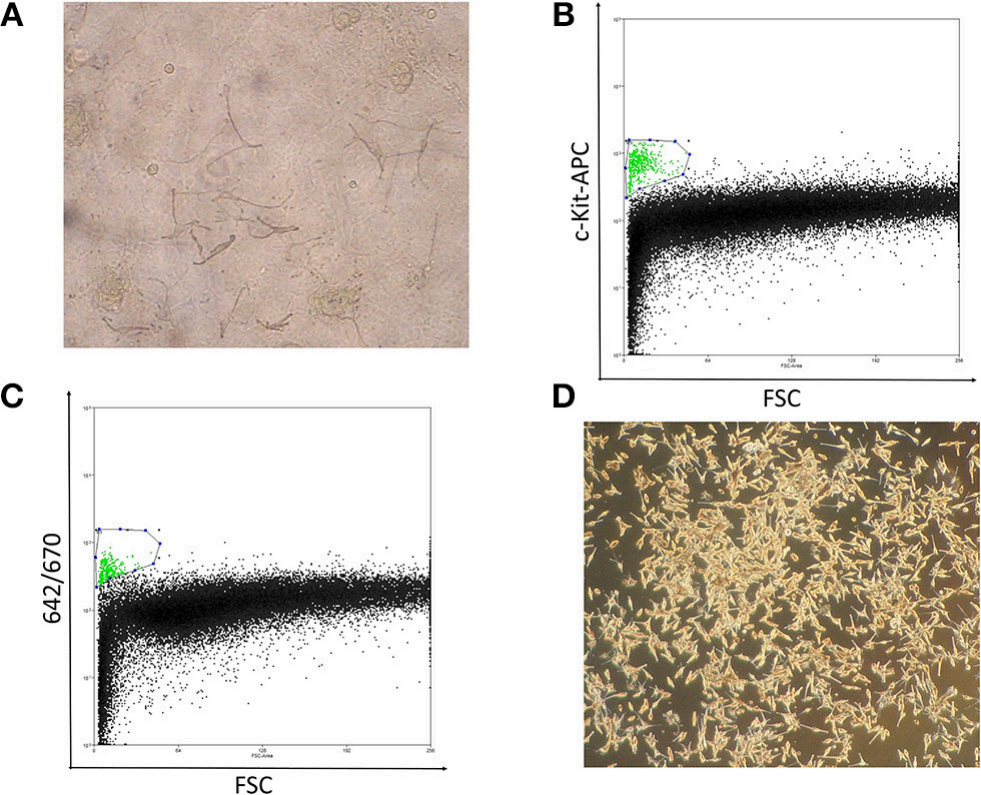
**(A)** Well-differentiated, dendritic melanocytes are present in the epidermal sheet. **(B)** Melanocyte sorting based on forward scatter and c-kit-APC staining intensity. The gated melanocyte population is highlighted in green. **(C)** Melanocyte sorting based on forward scatter and 642/670-nm excitation/emission. **(D)** Image of pure melanocytes cultured in DermaLife medium.

In preliminary studies, we attempted to obtain normal primary melanocytes from skin tissues with conventional single cell suspension cultures, however, the cells failed to propagate *in vitro*, possibly due to the donor ages. Alternatively, we employed an epidermal sheet cultivation technique using a feeder layer, in which melanocytes were cultivated alongside keratinocytes, which showed sufficient growth even with tissue from patients over 80 years of age (Figure 1A). For this, 1-cm^2^ normal skin specimens were surgically obtained from clear margins of resected melanoma tissue or normal abdominal skin, and cultivated to cell sheets ≥ 150 cm^2^ in culture flasks. Typically, after sheets of propagated cells were digested into a mixed, single-cell suspension, melanocytes should be isolated by flow cytometry using the antibody against c-Kit, melanocyte cell surface marker (14) (Figure 1B). However, to avoid artificial selection by c-kit methylation status, by isolating only c-kit positive melanocytes, we optimized an antibody-free sorting method in which a 642-nm semiconductor laser would excite some component of the melanocytes—likely melanin—that could be detected by a 670-nm detector (Figure 1C). The purity of melanocytes was confirmed based on the finding that the obtained cells expressed brown pigment and showed characteristic dendrites, and these cell populations showed almost the same dot plot as that of the cells obtained using c-kit (Figures 1A–D).

Notably, the combination of forward scatter and 670-nm emission was capable of purifying melanocytes from the mixed population. Although some keratinocyte and 3T3 feeder-cell contaminants were present, these cells are more resistant than melanocytes to trypsin dissociation, which enabled us to obtain a sufficiently pure melanocyte population. Significantly, these cell sorting methods allowed us to obtain the number of melanocytes necessary for epigenetic analysis with minimal environmental impact (Figure 1D).

### Clustering Analysis of DNA Methylation Profiles Revealed Strong Methylation Changes in Cultured Cells

**Figure 2:**
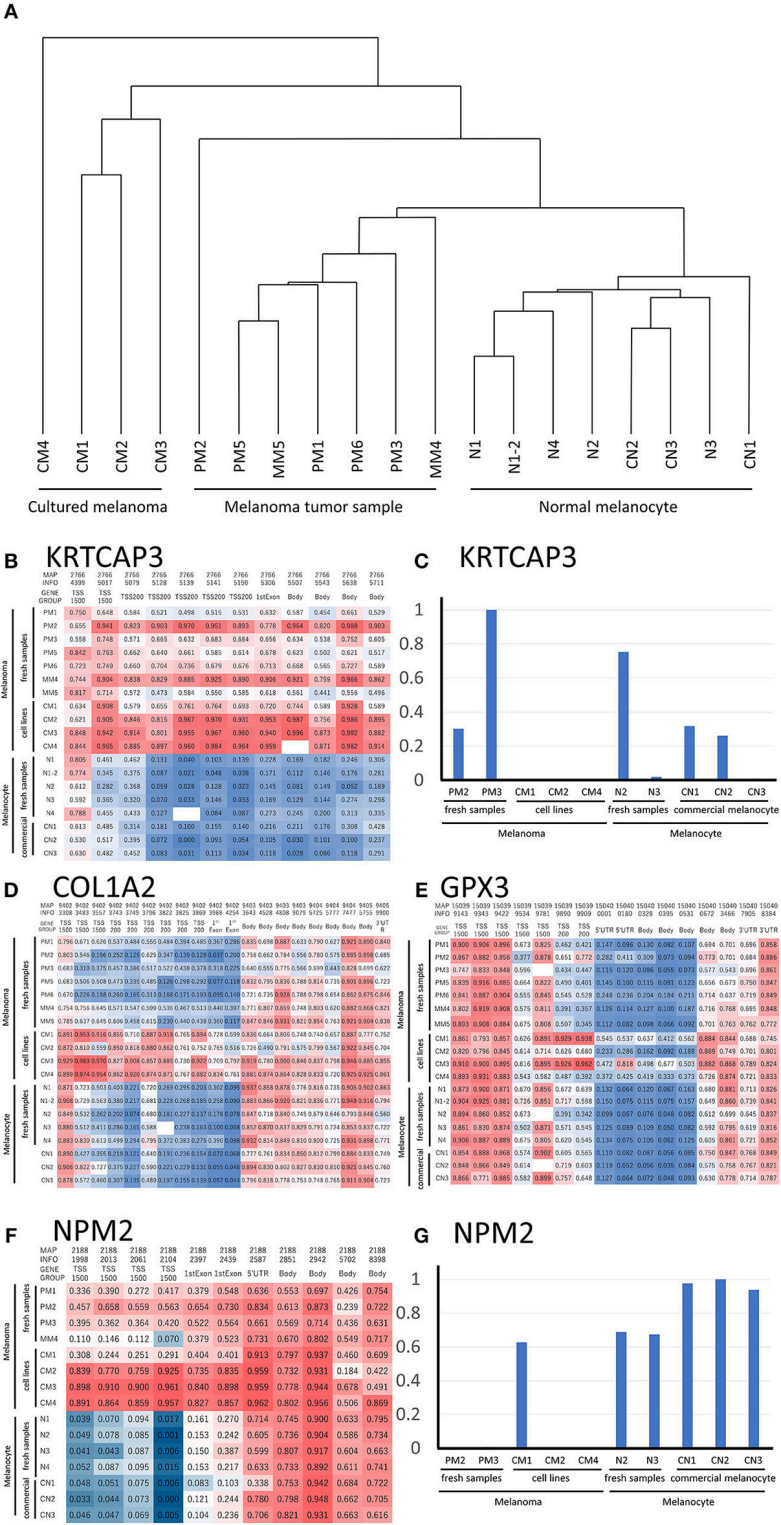
**(A)** Hierarchical clustering analysis of DNA methylation data from melanoma and melanocyte cell lines, and patient samples. **(B)** Methylation heat map of the 12 CpG sites in *KRTCAP3* for all 19 samples. The intermediate was defined as 50% of the methylation β value. **(D)** Methylation heat map of the 20 CpG sites in *COL1A2*. The intermediate was defined as 50% of the β value. **(E)** Methylation heat map of the 16 CpG sites in *GPX3* for all 19 samples. The intermediate was defined as 50% of the β value. **(F)** Methylation heat map of the 11 CpG sites in *NMP2* from the four melanoma/melanocyte paired tissue sets. The intermediate was defined as 20% of the β value. *KRTCAP3* **(C)** and *NPM2* **(G)** expression was examined in ten samples and normalized to that with the highest expression. TSS, transcription start site.

As shown in Table 1, we established sets of melanoma and melanocyte samples from four individuals. From Patient A, two melanocyte populations from different unaffected sites (Normal melanocytes N1 and N1-2) were obtained; a metastatic sample was obtained from Patient D (metastatic melanoma MM4). We also obtained primary and metastatic melanoma cells from a fifth patient, Patient E (Primary melanoma PM5 and metastatic melanoma MM5), and a primary melanoma from a sixth patient (PM6). In addition to these cells, we examined three commercially available normal human epidermal melanocytes (CN1–CN3) and four melanoma cell lines (CM1-CM4). DNA methylation status was examined in these 19 samples and analyzed by cluster analysis (Figure 2A). Notably, freshly isolated melanomas and melanocytes were clustered in the same group, whereas the four melanoma cell lines were placed in a separate clade, suggesting that long-term cultures or repeated freeze-thaw procedures may have an impact on methylation status. This finding clearly indicates the importance of using fresh melanoma samples, and not melanoma cell lines, in the analysis of methylation status. The commercially available normal melanocytes (CN1–CN3) were also clustered together with the normal melanocytes (N1–N4). Interestingly, the N1 and N1-2 melanocyte samples, obtained from different sites in the same individual, showed similar methylation patterns. Likewise, the PM5 and MM5 primary and metastatic melanoma samples also displayed similar methylation patterns. This result implies that there may be a subtle difference in the basal methylation state among individuals. Collectively, these data indicated that DNA methylation differs between melanocytes and melanomas. Although the present study does not have enough sample size and could not convincingly demonstrate the significance of comparing normal and malignant tissue from the same patients, the results indicate the importance of using sets of fresh melanocytes and melanoma samples for comparative methylation analysis. Volcano plots of *p*-values of each probe in global methylation vs. positive or negative percentage differences in methylation are shown in Figure S1. For hypermethylated CpG in melanoma, we chose the top 10 candidates from those with the lowest p-value and β value above 0.2, whereas for the hypomethylated CpG, we chose the top 5 candidates from those with the lowest p-value and β value < -0.4.

### Identification of Hypermethylated and Hypomethylated Genes in Melanoma Cells

We analyzed the differences in the methylation and gene expression between melanomas and melanocytes using four sets of menaocyte and melanoma samples obtained from the same individual. DNA hypermethylation generally results in gene downregulation (15). For each gene, average of β values was calculated for all CpG sites across a gene, including the transcription start site (TSS) and gene body. Table 2 shows the 10 genes with the highest methylation and 10 with the lowest methylation in melanoma samples. Our analysis revealed several novel genes that were hypermethylated in melanomas compared to melanocytes, including *KRTCAP3, AGAP2, ZNF490, TTC22*, and hypomethylated in melanomas compared to melanocytes, including *TDRG1, SDPR, GRIK2, GIMAP5, GPR31*, and *MIR548A2*. Of these, *KRTCAP3* (keratinocyte-associated protein 3) showed strong hypermethylation (11/12 CpG sites) in fresh melanoma samples and melanoma cell lines compared to that in melanocytes (Figure 2B). Interestingly, while hypermethylation reduced *KRTCAP3* mRNA expression in established melanoma cell lines, no significant downregulation was observed in fresh primary melanoma samples, PM2 and PM3, and melanocyte samples (Figure 2C).

### Comparative Methylation Analysis Showed Epigenetic Differences Between Freshly Isolated Cells and Propagated Cell Lines

We confirmed the previously reported hypermethylated and downregulated genes in melanoma in our samples, but we also found some differences between our analysis and previous reports. For example, Koga showed hypermethylation and limited expression of *COL1A2* in melanoma by using NimbleGen C4226-00-01promoter-tiling arrays and 2005-04-20_Human_60mer_1in2 genome wide human expression arrays (16). Chen reported hypermethylation and limited expression of *GPX3* in melanoma by using methylation specific PCR and qRT-PCR (5); however, while this was consistent with our analysis of melanoma cell lines, fresh samples showed no significant difference in methylation status (Figures 2D,E). These results suggest that prolonged culture and repeated freeze-thaw of melanoma cell lines induced changes in the DNA methylation status, thereby further supporting the preferential use of fresh specimens if possible.

### NPM2 Is a Potent Immunohistochemical Marker That Distinguishes Melanoma From Benign Melanocytic Lesions

NPM2 (nucleophosmin/nucleoplasmin 2) is a core histone chaperone involved in chromatin reprogramming. Our analysis indicated that the *NPM2* locus was far more hypermethylated in melanomas as compared to that in normal melanocytes. Although the average absolute difference in methylation of all 11 CpG sites was insignificant (β value/CpG site = 0.083) (Table 2), certain CpG sites—such as 21881998, 21882013, 21882061, and 21882104—showed remarkable differences between primary melanocytes and matched melanoma samples (Figure 2F); these four sites belonged to TSS 1500 gene group. Moreover, we confirmed the downregulation of *NPM2* mRNA expression in melanoma cells by expression microarray (Figure 2G) and real-time PCR (Figure S2), and the results were consistent with a previous report (16). Based on this, we hypothesized that NPM2 could be a clinical marker for diagnosing melanoma. For this, we first confirmed that all melanocytes and melanoma cells were stained with the phenotypic melanocye marker Melan-A (Figures S3a,b). Immunostaining with NPM2 antibody showed clear positive findings in the nucleus of melanocytes, but nuclei of malignant melanoma cells showed no staining (Figures S3a,b). As shown in Tables 3A,B, NPM2 was observed in only five melanoma samples (15.6%, 5/32; *P* = 0.001) while most of the archived melanocyte samples displayed NPM2 staining (74.6%, 50/67). When compared between melanoma and benign nevus (23.8%, 10/42; *P* = 0.39) in all lesions including both the epidermis and dermis, no clear difference was observed (Table 3A). On the contrary, NPM2 staining was observed in Clark's nevi with only intraepidermal lesion (80%, 8/10), and there was a significant difference compared to that in malignant melanoma *in situ* (15.6%, 4/14; *P* = 0.013). Thus, negative NPM2 immunostaining may be an effective clinical marker to distinguish melanomas from benign melanocytic lesions, and may be a target for epigenetic manipulation in the future.

## Discussion

For epigenetic analysis in cancer, it is preferable to compare cancer and normal cells from the same patient because epigenetic status may be altered by environmental factors. Since ultraviolet irradiation is one of the environmental factors that can change the methylation state, the methylation status of melanomas at the two types of sun-exposed sites (PM1 and PM2) and two sun-shielded site (PM3 and MM4) was analyzed. However, no clear trend was found, probably because the number of samples was small and the difference in methylation between individuals was large (Figure S5). In this study, we attempted to cultivate primary melanocytes from normal skin tissue specimens collected from melanoma patients; however, while melanocytes isolated from young subjects proliferated easily, those from elderly patients (over 70 years of age) showed limited growth. This is consistent with a previous report that demonstrated that melanocytes from individuals over 30 years of age are difficult to propagate in culture (9). Since most patients with malignant melanoma are middle-aged or older, a new melanocyte cultivation method was considered in our study. In the present study, melanocytes were propagated continuously with an epidermal sheet culture technique. This method is likely to be advantageous since the surrounding keratinocytes and fibroblasts play an important role in the proliferation of melanocytes. Moreover, since epidermal sheet culturing may also affect the methylation status, samples were cultured for a minimal period, necessary for analysis.

Cell sorting usually requires fluorescent antibodies against surface antigens. The receptor tyrosine kinase, c-Kit, is a common melanocyte marker used for FACS analysis; however, antibody ligation may result in selection depending on the c-kit expression. Therefore, we examined several excitation laser/detector combinations with a MoFlo XDP flow cytometer and found that analysis at 642/670 nm was capable of separating a nearly pure melanocyte population without using antibodies.

Most epigenetic studies on melanoma have compared samples from different individuals, wherein the results could by affected by the age and other environmental circumstances of the donor. Interestingly, our analysis of *COL1A2* and *GPX3*—previously reported to be hypermethylated in melanoma—in the four patient-derived sets of melanoma and melanocytes showed completely different results. The main reason for this result may be that prolonged culture and repeated freeze-thaw of melanoma cell lines induces changes in the DNA methylation status. In addition, it could be possible that the use of fresh melanoma tissue samples influenced the methylation status because stromal and inflammatory cells surrounding the tumor tissue could have partially modified the methylation state. Moreover, genes showing hypermethylation in melanoma such as *KRTCAP3, AGAP2, ZNF490*, and *TTC22* were newly recognized in the present study. However, although *KRTCAP3* was highly methylated, there was no clear difference in mRNA expression (Figure 2G). Furthermore, expression of *AGAP2, ZNF490*, and *TTC22* was not decreased in melanoma (data not shown). In general, DNA methylation inhibits the binding of transcription factors, thereby down-regulating gene expression; however, some transcription factors have been shown to bind to methylated sites (17). Thus, the association between DNA methylation and gene expression remains contentious.

In this study, comparisons between melanoma samples and melanocyte samples were made through the averaged methylation level of CpGs across each gene or methylation status of all individual CpGs. Analysis was also carried out for the region groups of TSS 1500, TSS 200, 5′UTR, 1st exon, gene body, 3′UTR or N Shelf, N Shore, CpG Island, S Shore, S Shelf, as classified by the Illumina chip. There was no clear trend between the methylation status of CpGs in each region and the gene expression (some of them are shown in Figure S4). However, in NPM2, there was a correlation between expression and methylation in the TSS 1500 region. Even if significant differences were not found in the analysis of average CpG methylation across a gene, there is a possibility that significant information may be found in the methylation status of individual CpGs. In the current study, the number of samples was insufficient for such an analysis; moreover, CpGs that can be measured with Illumina Human Methylation 450 BeadChip are also limited. Therefore, more comprehensive analysis using a larger number of samples is needed in the future.

**Figure 3:**
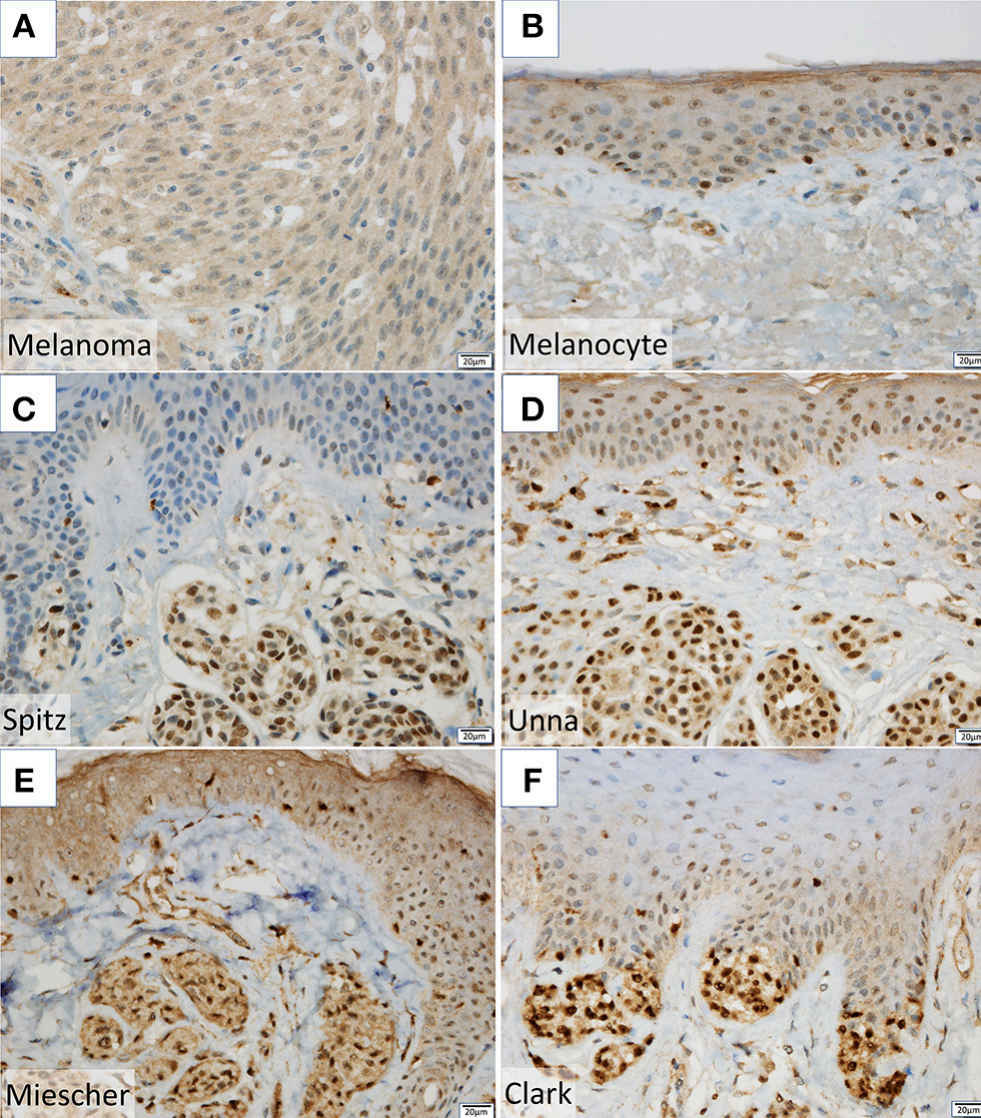
**(A,B)** NPM2 expression was evaluated in melanoma and normal melanocytes in the basal epidermal layer by immunohistochemistry. **(C–F)** NPM2 immunostaining for the following benign nevi: **(C)** Spitz, **(D)** Unna, **(E)** Miescher, and **(F)** Clark.

It is often difficult to distinguish between benign melanocytes and malignant melanoma cells. Routine pathological examination typically consists of hematoxylin and eosin staining, and supplemental staining with Melan-A, HMB-45, and SOX10 (18), which often yields vague differences in staining intensity. Therefore, we attempted to utilize new genes as diagnostic markers, by analyzing highly methylated and downregulated genes. In this study, we found a significant difference in NPM2 staining property between normal pigment cells and malignant melanoma cells (Figures S3a,b). Although there was no obvious difference from a benign melanocytic nevus lesion in the dermis, it was possible to distinguish between malignant melanoma *in situ* and intraepidermal Clark's nevi, which is the most problematic in daily clinical diagnosis (Figures 3C–F, Table 3). While this result supports the use of NPM2 as a molecular marker of normal tissue, further studies with a larger study population would be required before its clinical application. It is suggested that NPM2 levels may decrease with malignant transformation and it may be involved in the early events in the development of malignant melanoma. Further analyses may reveal NPM2 as a potential therapeutic target in the future.

In this study, new findings on DNA methylation in malignant melanoma were presented, but interpretation of the results would need more attention, because the samples of malignant melanoma used were of mixed subtypes and were limited in number, and the DNA methylation landscape of melanoma is heterogeneous in nature.

In conclusion, the present study developed an epidermal sheet culture and cell sorting method to obtain an enriched population of melanocytes for use in epigenetic profiling studies. Candidate epigenetic alterations were compared with those from previous reports to identify meaningful differences in gene methylation with potential of being diagnostic markers or therapeutic targets.

## Author Contributions

SF, HN, and CN designed the project and wrote the manuscript. SF, HN, HJ, NJ, TT, and MI performed experiments. SF and HJ performed statistical data evaluation.

### Conflict of Interest Statement

The authors declare that the research was conducted in the absence of any commercial or financial relationships that could be construed as a potential conflict of interest.

## Supplementary Material

Figure S1 Volcano plots of *p*-values of each probe in global methylation on Y-axis vs. positive or negative β value differences in methylation (4 pairs of melanoma minus melanocyte) on X-axis are shown. Hypermethylated CpGs, 10 CpG plots with lowest *p*-value and β value above 0.2; hypomethylated CpGs, 5 CpG plots with lowest *p*-values and β value < -0.4 are listed.

Figure S2 *NPM2* mRNA expression was analyzed by real-time PCR. Normalization was performed using GAPDH. The bars represent mean + SEM (*n* = 2).

Figure S3 Melan-A expression was evaluated in the basal epidermal layer of **(a)** melanoma and **(b)** normal melanocytes by immunohistochemistry.

Figure S4 Difference in methylation β value (4 pairs of melanoma minus melanocyte) was plotted on the X-axis and the logarithmic axis of the gene expression ratio on the Y-axis. **(a)** All CpGs, **(b)** CpG groups in the TSS 1500 region, and **(c)** CpG groups in the TSS200 region were plotted.

Figure S5 Hierarchical clustering analysis of DNA methylation data from two sun-exposed melanoma (PM1 and PM2) and two sun-shielded melanoma (PM3 and MM4) samples.
