# Supplementary figures and images for: Gene Expression and Methylation Analysis in Melanomas and Melanocytes From the Same Patient: Loss of NPM2 Expression Is a Potential Immunohistochemical Marker for Melanoma
Source: Front Oncol. 2019 Jan 21;8:675. doi: 10.3389/fonc.2018.00675 (PMC6348333; doi:10.3389/fonc.2018.00675)

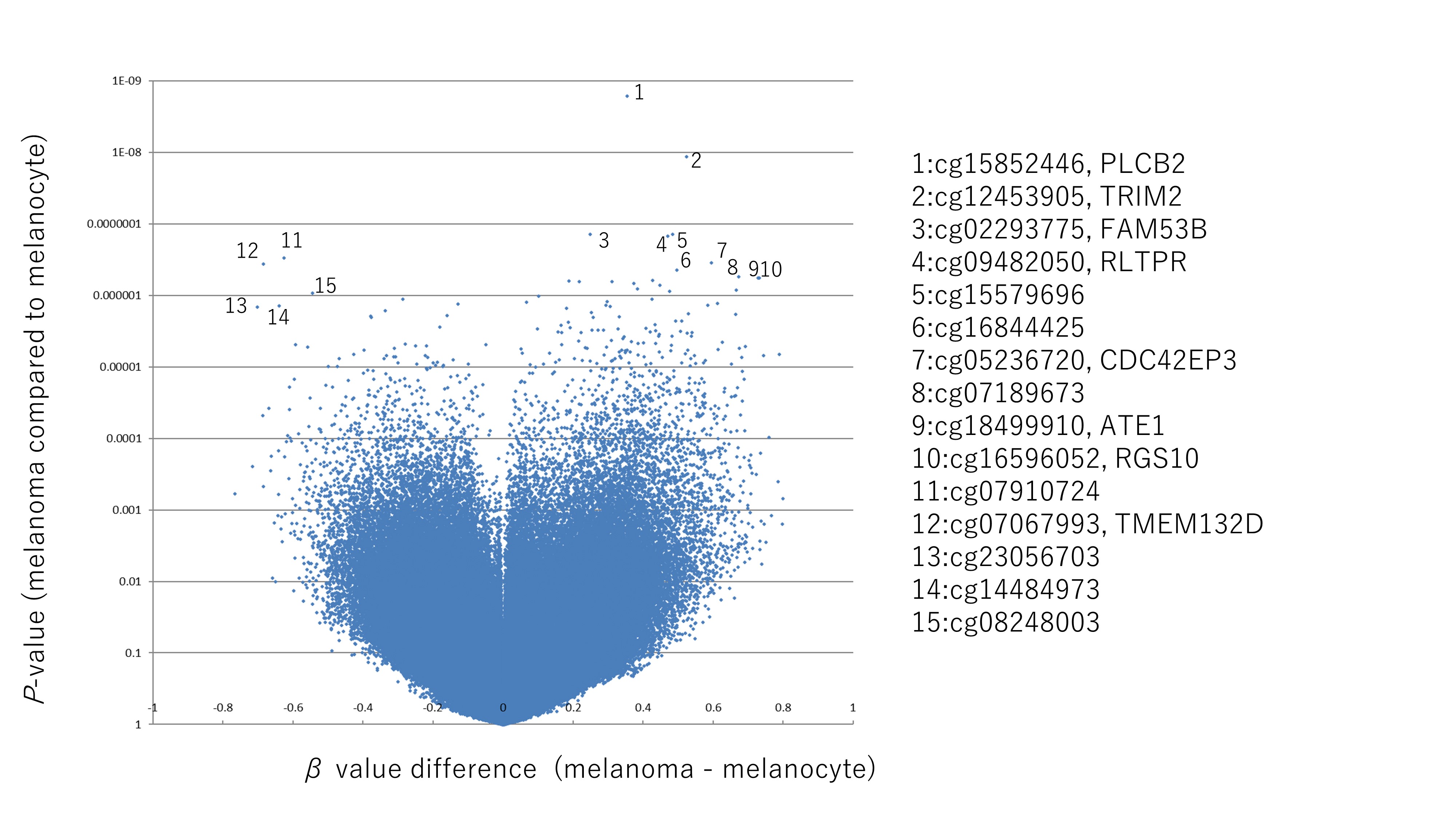

Supplement: Figure S1 — Volcano plots of p-values of each probe in global methylation on Y-axis vs. positive or negative β value differences in methylation (4 pairs of melanoma minus melanocyte) on X-axis are shown. Hypermethylated CpGs, 10 CpG plots with lowest p-value and β value above 0.2; hypomethylated CpGs, 5 CpG plots with lowest p-values and β value < -0.4 are listed. [file Image_1.JPEG]

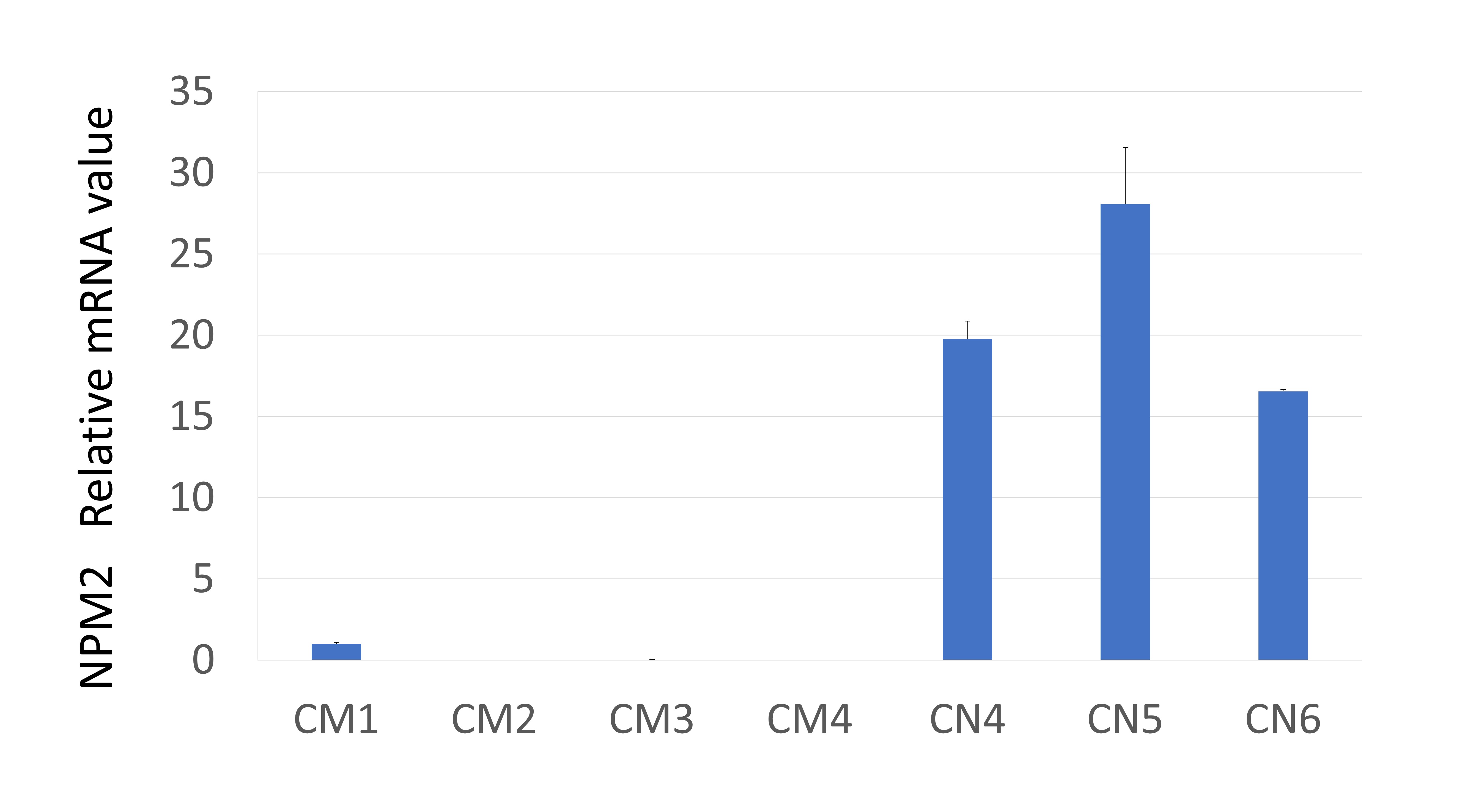

Supplement: Figure S2 — NPM2 mRNA expression was analyzed by real-time PCR. Normalization was performed using GAPDH. The bars represent mean + SEM (n = 2). [file Image_2.JPEG]

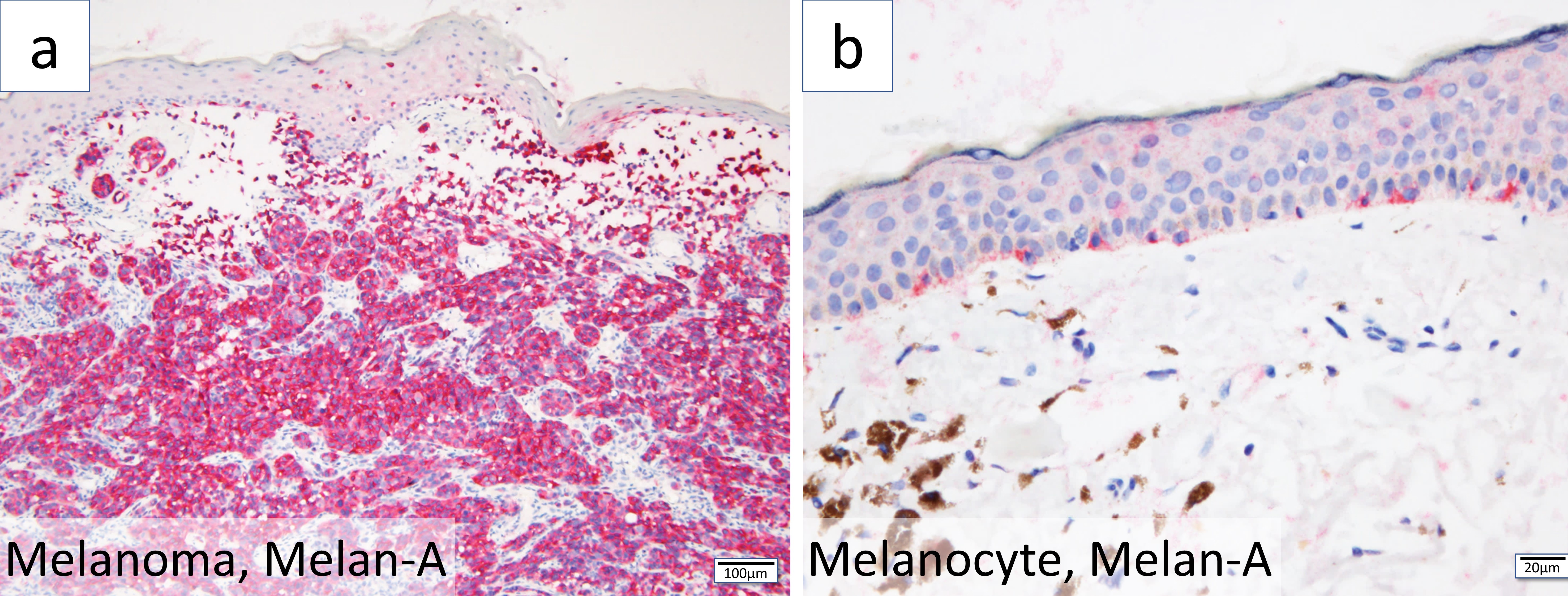

Supplement: Figure S3 — Melan-A expression was evaluated in the basal epidermal layer of (a) melanoma and (b) normal melanocytes by immunohistochemistry. [file Image_3.JPEG]

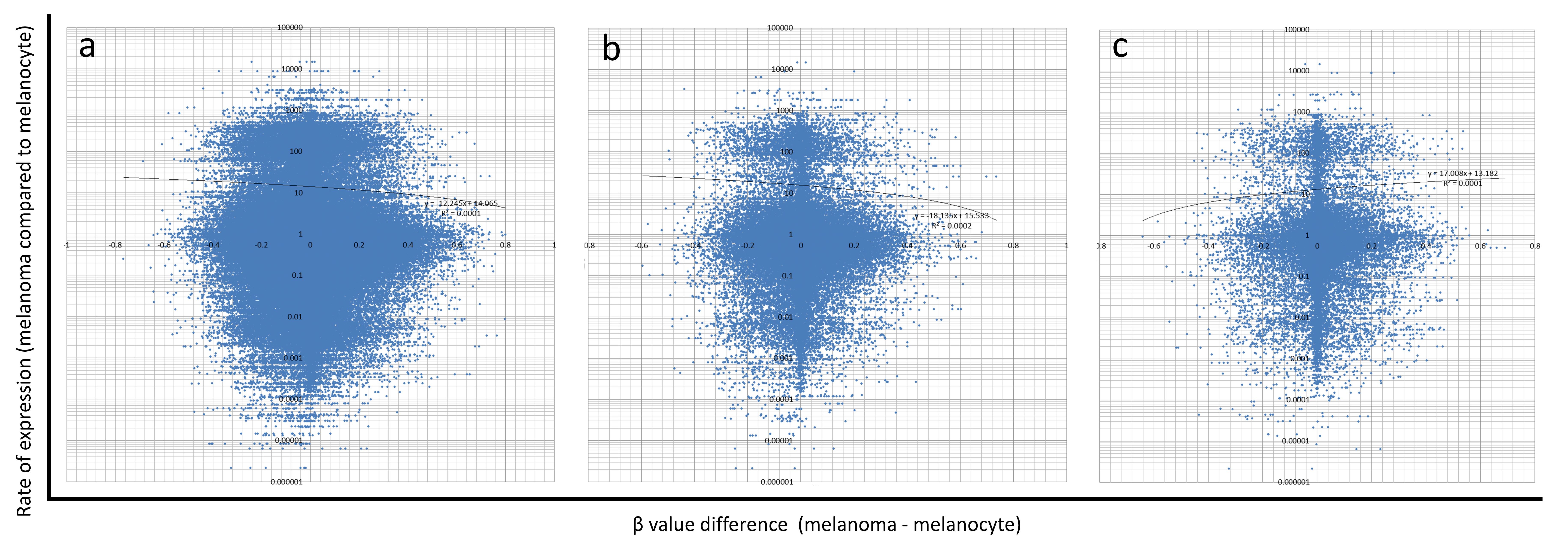

Supplement: Figure S4 — Difference in methylation β value (4 pairs of melanoma minus melanocyte) was plotted on the X-axis and the logarithmic axis of the gene expression ratio on the Y-axis. (a) All CpGs, (b) CpG groups in the TSS 1500 region, and (c) CpG groups in the TSS200 region were plotted. [file Image_4.JPEG]

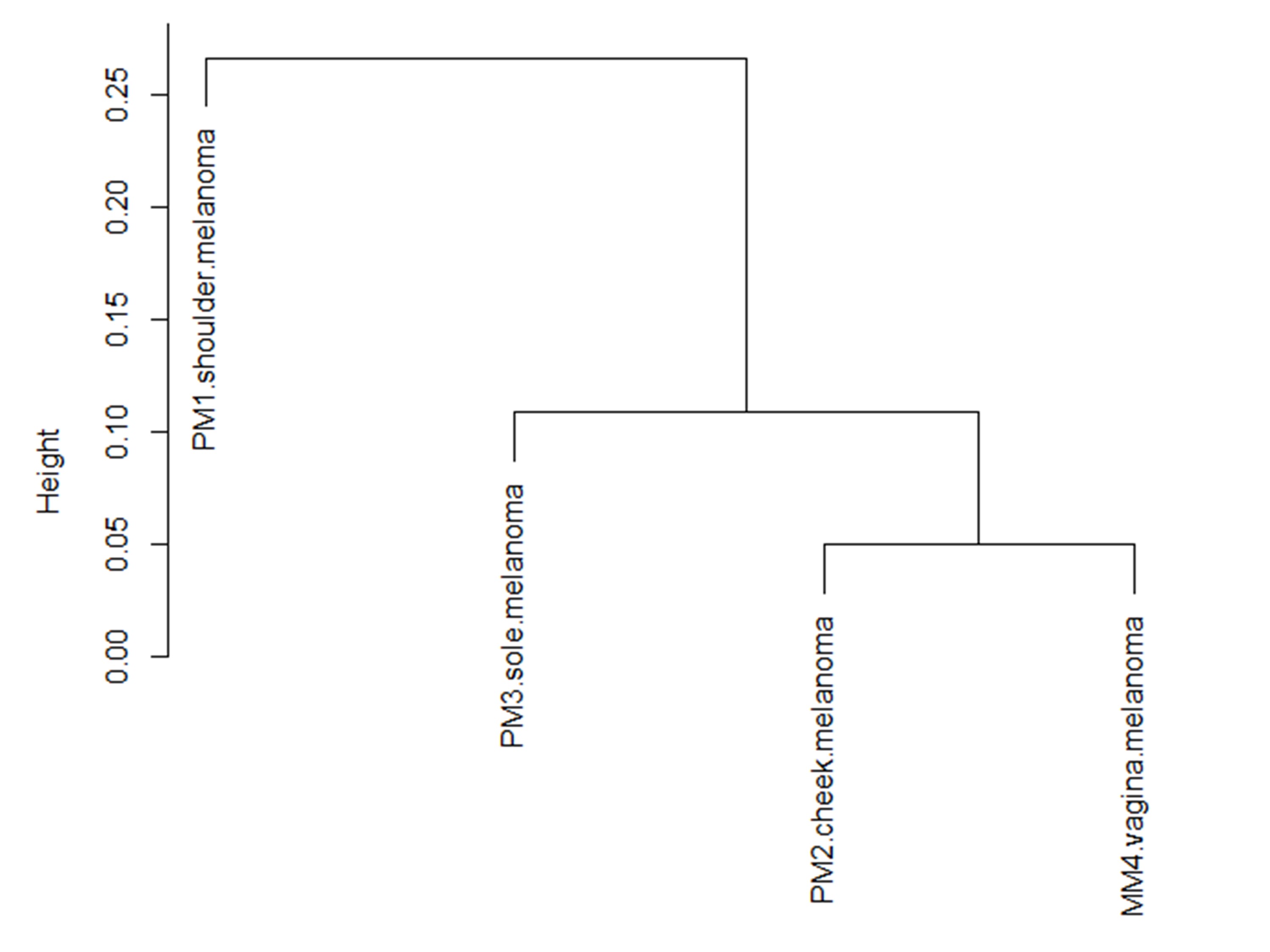

Supplement: Figure S5 — Hierarchical clustering analysis of DNA methylation data from two sun-exposed melanoma (PM1 and PM2) and two sun-shielded melanoma (PM3 and MM4) samples. [file Image_5.JPEG]
